# Phenology and Breeding Mechanisms of *Calamus lobbianus* Becc. and *Calamus pygmaeus* Becc

**DOI:** 10.21315/tlsr2024.35.3.9

**Published:** 2024-10-07

**Authors:** Ng Win Seng, Wong Sin Yeng, Hashimah Elias, Meekiong Kalu

**Affiliations:** 1 Institute of Biodiversity and Environmental Conservation, Universiti Malaysia Sarawak, Jalan Datuk Mohammad Musa, 94300 Kota Samarahan, Sarawak, Malaysia; 2 Harvard University Herbaria, 22 Divinity Avenue, Cambridge, MA 02138, United States of America; 3 Faculty of Resource Science and Technology, Universiti Malaysia Sarawak, Jalan Datuk Mohammad Musa, 94300 Kota Samarahan, Sarawak, Malaysia

**Keywords:** Breeding Mechanism, Floral Rewards, Floral Visitors, Operational Sex Ratio, Phenology, Fenologi, Ganjaran Bunga, Mekanisme Pembiakan, Nisbah Jantina Beroperasi, Pelawat Bunga

## Abstract

The floral biology of *Calamus* is relatively unknown except for several species. In this study, *Calamus lobbianus* and *Calamus pygmaeus* were selected to represent the non-climbing rattan of the Sundaland’s flagellate group. Observations on phenology, floral rewards and floral visitors as well as experiments on the breeding mechanism and operational sex ratio were performed for both species. For both species, anthesis started in the early morning for pistillate and staminate plants, ended by late noon (staminate plants) but lasted till the next day in pistillate plants for both species. Although both species appeared to be aseasonal in flowering, *C. lobbianus* exhibited a male-biased population while *C. pygmaeus* did not exhibit any sex bias. Nectar was observed to be extruded from the base of the petals of *C. lobbianus* of the staminate flowers and sterile staminate flowers of the pistillate plants. The concentration and volume of the nectar of the staminate flower and sterile staminate flower of *C. lobbianus* peaked at c. 11% and 9 μL around 1100 (Day 1) and c. 13% and 8 μL around 0930 (Day 1), respectively, but only appeared as a layer of glistening exudate in *C. pygmaeus*. Floral scent was not detected in any of the inflorescences of both species. Several insect visitors were observed to be the primary visitors of both *Calamus* species which include two species of *Tetragonula*, a species of *Liostenogaster* sp., and *Stenodyneriellus* sp. Experiments on breeding mechanism of *C. lobbianus* and *C. pygmaeus* indicate that both species are most likely apomictic. *C. pygmaeus* is capable of vegetative propagation through the rooting at the tip of inflorescence.

## Abstract (fr)

**Abstrak:**

Biologi flora *Calamus* kurang difahami kecuali untuk beberapa spesies. Dalam kajian ini, *Calamus lobbianus* dan *Calamus pygmaeus* telah dipilih untuk mewakili rotan tidak memanjat dalam kumpulan berflagelum di Sundaland. Pemerhatian fenologi, ganjaran bunga dan pelawat bunga serta eksperimen mekanisma pembiakan dan nisbah jantina beroperasi telah dijalankan bagi dua spesies tersebut. Antesis bagi pokok betina dan jantan bermula dari awal pagi dan berakhir lewat petang (pokok jantan) manakala antesis bagi pokok pistilate berlangsung sehingga hari seterusnya. Pemerhatian pembungaan bagi kedua-dua spesies adalah tidak bermusim tetapi pembungaan *C. lobbianus* mempamerkan bias ke populasi jantan manakala *C. pygmaeus* tidak mempamerkan bias terhadap kedua-dua jantina. Nektar hanya dihasilkan pada bahagian dasar kelopak bunga jantan dan bunga jantan steril diad di sebelah bunga betina *C. lobbianus*. Pengeluaran nektar bunga jantan *C.lobbianus* memuncak sekitar jam 1100 (hari pertama) dengan kepekatan 11% dan isipadu sebanyak 9 μL. Pengeluaran bunga jantan steril memuncak sekitar jam 0930 (haripertama) dengan kepekatan 13% dan isi padu sebanyak 8 μL manakala *C. pygmaeus*hanya menghasilkan lapisan cecair berkilat. Bau bunga kedua-dua spesies tidak dapatdikesan pada jambak bunga. Dua spesies *Tetragonula* dan satu spesies *Liostenogaster*sp. diperhatikan sebagai pelawat utama untuk kedua-dua spesis *Calamus*. Eksperimenterhadap mekanisma pembiakan menunjukan bagi kedua-dua spesies *Calamus* adalahberkemungkinan apomik. *C. pygmaeus* mempunyai keuapayaan untuk menjalankanpropagasi secara vegetatif melalui penakaran pada hujung jambak bunga.

**Highlights**
- Anthesis of *Calamus lobbianus* lasts about 9 h and 31 h for staminate and pistillate flowers respectively while anthesis of *Calamus pygmaeus* lasts about 6 h and 25 h for staminate and pistillate flowers, respectively.
- Both forms of *C. lobbianus* secrets nectar on Day 1 of anthesis for around four hours each from early morning towards noon.
- Two species of *Tetragonula*, one species of *Liostenogaster* sp. and one species of *Stenodyneriellus* sp. was observed to be the primary visitors of both *Calamus* species.

## INTRODUCTION

*Calamus* L. currently comprises ca. 520 species which now also include *Ceratolobus* Blume, *Daemonorops* Blume ex Schult.f., *Pogonotium* J. Dransf. and *Retispatha* J.Dransf. sensu Baker (2015) while Henderson (2020) recognises 411 phylogenetic species out of 516 sampled species*. Calamus* is distributed across six main regions (Afro-India, Indo-Burma, Philippines, Sundaland, Wallacea and Sahul with Borneo alone having 111 species) and divided into distinct groups where only a few species are widespread over three or more regions (Henderson 2020). Several *Calamus* species stand out with certain unique features such as the following three species where the inflorescence apices can develop into new plants, e.g., *Calamus pygmaeus* Becc., *C. castaneus* Griff. (Ruppert *et al*. 2012), and *C. kampucheaensis* A. J. Hend. & Hourt (Henderson 2020). Even the flagella apices can develop into new plants, as in *C. gamblei* Becc. which appears to be common within the species (Renuka & Nambiar 1985). *C. pygmaeus* is placed in a group of six other flagellate species that occur within Sundaland (*C. comptus* J.Dransf., *C. gaharuensis* A. J. Hend, *C. lengguanii* A. J. Hend, *C. nematospadix* Becc., *C. nielsenii* J. Dransf. and *C. psilocladus* J. Dransf.) (Henderson 2020).Several characters that members of this group shared are the reddish-brown indumentum along rachis and pinnae base and small whitish globose fruits with explanate fruiting perianths and scarcely developed knees (absent in *C. pygmaeus*)(Henderson 2020).

*C. lobbianus* Becc. belongs to the *C. conirostris* Becc. (a subgroup of the Sundaland flagellate group) group consisting of seven members (*C. conirostris, C. convallium* J. Dransf., *C. gonospermus* Becc., *C. leloi* J. Dransf., *C. lobbianus, C. pycnocarpus* (Furtado) J. Dransf. and *C. spectatissimus* Furtado). This group is delimitated by having the rachis of the partial inflorescence thicker than the rachis of the inflorescence distal to the partial inflorescence. Members of the group also tend to have brown or black fruits with non-channelled scales (Henderson 2020).

A better understanding of the genus’ floral biology and pollination mechanisms may allow better classification for a genus of this magnitude as up until 1986, the leading view in the scientific literature was that palms were mainly anemophilous (Henderson 1986). The floral biology of *Calamus* can be considered understudied relative to the size of the genus; species investigated so far included *C. caesius* Blume, *C. castaneus, C. longisetus* Griff., *C. manan* Miq., *C. peregrinus* Furtado, *C. rudentum* Lour., *C. subinermis* H.Wendl. ex Becc. and several species previously belonging to *Ceratolobus* (Alloysius 1999; Bøgh 1996; Dransfield 1979a; 1979b; Kidyoo & McKey 2012; Lee 1995; Mohd Rusdi *et al*. 2022). Flowers with either nocturnal (Alloysius 1999; Bøgh 1996) or diurnal anthesis (Kidyoo & McKey 2012; Lee 1995). The phenology of both staminate and pistillate flowers is similar across the genus. In the staminate flowers, anthesis begins with the opening of sepals and petals followed by the anthers emerging to release pollen and the flower dehisces soon after. In the pistillate flowers, the anthesis is initiated by the stigma emerging till the tip of the stigma is recurved and points towards the base of the flower which then slowly turns brown towards the end of the anthesis (Alloysius 1999; Bøgh 1996; Kidyoo & McKey 2012). *Calamus* lacks distinct features to attract specific pollinators but tends to be visited mostly by Hymenopterans such as *Apis* and *Trigona* which are potential pollinators but may sometimes act as pollen thieves as well (Alloysius 1999; Bøgh 1996; Dransfield 1979a; 1984; Kidyoo & McKey 2012). Moths have been observed to be potential pollinators during nocturnal visits (Alloysius 1999; Lee 1995). Most species of *Calamus* previously placed in *Calospatha* Becc., *Ceratolobus* and *Daemonorops* were recorded to emit a musty floral odour and were visited by many beetle species which are potential pollinators (Dransfield 1979a).

Studies on the floral biology of *Calamus* covered several groups including the groups of *C. albidus* L. X. Guo & A. J. Hend*., C. arborescens* Griff. and *C. arugda* Becc., in either its natural habitats or plantations (Alloysius 1999; Bøgh 1996; Hj Abdullah 2000; Kidyoo & McKey 2012; Kitching *et al*. 2007; Lee 1995; Mohd Rusdi *et al*. 2022), however, none has been conducted so far on the groups represented by *C. lobbianus* and *C. pygmaeus. C. lobbianus* and *C. pygmaeus* while similar in phenology, exhibit stark differences in their floral characteristics and attractants, leading to distinct reproductive strategies. This contrast will directly be reflected on the visitor assemblage which subsequently should reveal their breeding strategy. Therefore, this study aims to investigate the floral biology and the pollination mechanism of two non-climbing species, *C. lobbianus* and *C. pygmaeus*.

## METHODOLOGY

### Study Site

This study was conducted at Kubah National Park (KNP) (1°36′45.51″N, 110°11′48.52″E) which is approximately 20 km northwest of Kuching, Sarawak, Malaysia. Populations of *C. lobbianus* and *C. pygmaeus* occur between 200 m– 500 m elev. Kubah National Park comprises largely five vegetation types: lowland mixed dipterocarp forest, kerangas forest, submontane forest, alluvial forest and secondary forest (Pearce & Geri 2007). Red-yellow podzolic soils cover most of the rocks in KNP, dip-slope podzols (predominantly sandy clay, well-drained and nutrient deficient) and peaty soil are common on exposed ridges upward from about 300 m elev. (Hazebroek & Kashim 2001). Kubah National Park faces the Southwest Monsoon (late May–early October) and the Northeast Monsoon (late November–late March) with drier weather intervals between April to May and October to November. The average rainfall in the Kuching–Matang area is c. 4200 mm in the year 2020 (Jabatan Meteorologi Malaysia 2020). Pearce (1992) reported that KNP has the highest diversity of palms in Sarawak with at least 95 taxa of palm species and at least 47 species of *Calamus* within the park and its immediate environs.

Plant individuals were sampled on both sides along the main trail and Selang trail. Individuals were selected based on the presence of senesced inflorescence or infructescence from the previous season. The duration of the whole study lasted from April 2021 to April 2022 (Appendix A). The number of individuals and flowering individuals were noted. Individuals of *C. pygmaeus* with rooted individuals were noted along the sampling trail. The voucher specimens of both species including the rachillae of both staminate and pistillate partial inflorescences were collected, fixed in alcohol and deposited at Sarawak Herbarium (SAR).

### Species

**Figure 1:**
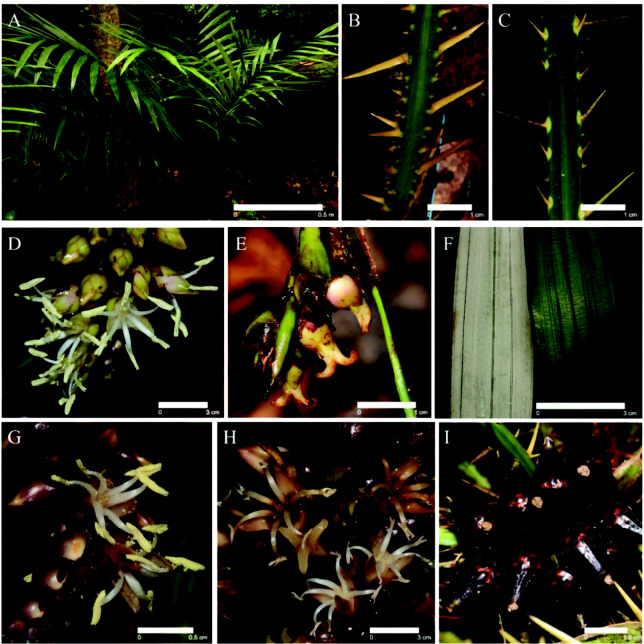
(A) *Calamus lobbianus*, acaulescent up to 2 meters tall; (B) Stouter irregularly arranged spines on petiole of Morph A; (C) Thinner irregularly arranged spines on petiole of Morph B; (D) Greenish pink staminate flowers of Morph A; (E) Greenish pink pistillate flowers of Morph A; (F) Powdery white abaxial and glabrous adaxial leaves of *C. lobbianus* leaves; (G) Maroon staminate flowers of Morph B; (H) Maroon pistillate flowers of Morph B; and (I) Mature infructescence bunch with top-shaped fruits covered in black and brownish-yellow scales. Scale bars are included.

**Figure 2:**
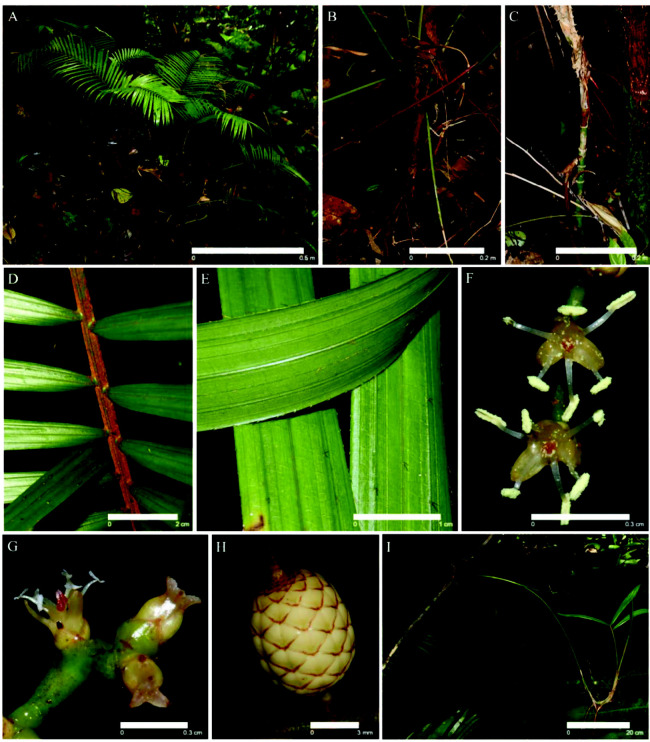
(A) *Calamus pygmaeus* up to 1.5 m tall; (B) Acaulescent stem with brown sheaths and green stout spines; (C) Reddish-brown indumentum along the rachis; (D) Black soft spines along the adaxial, abaxial and pinnae margin; (E) Staminate in bloom with anthers covered in powdery yellow pollen and bright orange pistillode; (F) Staminode flower in bloom with tri-lobed stigma protruding from the pistillate flower; (G) Globose fruit white scales and brown margins; and (H) Tip of inflorescence rooting into a new plantlet with the remnant of partial inflorescence still attached. Scale bars are included.

*Calamus lobbianus* (Fig. 1) is a solitary stemless or very short-stemmed and sometimes subterranean, up to 2.0 m tall whereas *C. pygmaeus* (Fig. 2) is a clustered rattan, acaulescent up to 1.5 m tall. The inflorescences of both pecies have up to six first-order branches (partial inflorescences) distributed along the main axis (terminates with a flagellum in *C. pygmaeus*) An individual stem could produce up to four inflorescences in a single flowering season. In the staminate inflorescences, the flowers are alternately and distichously arranged along the rachillae. The rachillae of pistillate inflorescences bear flowers in dyads which consist of a pistillate flower and a sterile staminate flower.188

*C. lobbianus* occurs on soil high in organic matter in damp facies of hill forests (Fig. 1A). Most of the individuals were found near the streams under shade and sometimes in open areas with underexposed sunlight. The stem reaches c. 0.6 m long with dull green sheaths which are not tubular and open opposite the petiole. The sheath is densely armed with triangular spines which are concave at the base proximally, horizontally spreading, yellowish-green and 5 mm–50 mm long (Figs. 1B and 1C). The knee, ocrea and flagellum are not present in any of the individuals in the population. The leaf is ecirrate, c. 140 cm with the petiole up to 82 cm long, armed laterally with yellowish-green horizontal spines, c. 26 mm and rachis c. 30 cm long. The leaflets about 20 on each side of the rachis, are regularly arranged but rather distant c. 40 cm × 3 cm. The leaflets are lanceolate, with spinules on veins abaxially, the lateral veins approximately parallel, “broken” vein dark green on the adaxial surface, densely chalky-white on the abaxial surface and apical leaflets very briefly joined (Fig. 1F). The inflorescence is short and slender, c. 42 cm long in staminate and 27 cm long in pistillate plants. The peduncle is scarcely developed above the point of divergence. The prophyll is tubular and persistent, usually splitting only at the apex. The partial inflorescences are not stalked, with a pulvinus between the axil of the rachis and the partial inflorescence, with the rachis markedly thicker than the inflorescence rachis distal to the partial inflorescence. The rachis of the bracts is tubular and somewhat inflated at the apex, with an oblique opening and a sessile rachilla. The partial inflorescences of both sexes branch into two orders. The partial pistillate and staminate inflorescence are c. 11.5 cm long and 18.2 cm long, respectively. The staminate sepals are usually shorter than the petals, cupular, trilobed at the apex, large, fleshy and bi-coloured. The stamens are six with uniseriate filaments, inflexed at the apex producing light-yellow powdery pollen. The associated pistillode is opaque and trilobed. The pistillate inflorescences comprised of sessile dyads of a pistillate and sterile staminate flower. The calyx, petal and stigma are trilobed. The fruit is top-shaped, c. 34 mm × 18 mm diameter with the fruiting perianths explanate and covered in c. 15 vertical rows of brownish black, glossy and smooth scales (Fig. 1I). Two morphologically different forms of *C. lobbianus* were found [Form A (Figs. 1D and 1E) and Form B (Figs. 1G and 1H)] with variations in the spines of the petiole and flowers of both sexes. The vegetative and reproductive features of both forms in staminate and pistillate plants of *C. lobbianus* are presented in Table 1.

Populations of *C. pygmaeus* were mostly found in well-irrigated sandy clay soil with a top layer of organic matter (Fig. 2A). At least two populations were found by a stream. The leaf-sheaths of *C. pygmaeus* are not tubular but open opposite the petiole, dull brown, bearing rather few groups of horizontal or slightly reflexed triangular green stout spines, c. 6.1 mm with abundant brown indumentum between spines (Fig. 2B). The flagellum and knee are absent while the ocrea is short, membranous and soon tattering. The leaf is ecirrate, c. 75.5 cm long with the petiole c. 21.5 cm, armed with whorls of horizontal spines near the base but unarmed distally, sparsely covered with reddish-brown or brown indumentum and the rachis (Fig. 2C). The leaflets are c. 28 on each side of the rachis, c. 33.5 cm long, unarmed, regularly arranged, linear, with spinules on veins adaxially, the lateral veins approximately parallel, one terminating subapically, leaving a distinct or obscure, adaxial “broken” vein, the bases abaxially sparsely covered with black indumentum (Fig. 2D). The inflorescence emerges between the leaves, slender and whip-like up to c. 2.5 m long in pistillate plant and c. 2.7 m long in the staminate plant, peduncle c. 70 cm long in both staminate and pistillate plants both roots at the apex to generate new plant (Fig. 1H). The partial inflorescences (up to four) of the pistillate plant and staminate plant are c. 5.7 cm long and c. 6.2 cm long, respectively, which are spread sparsely along the inflorescence, stalked, with a pulvinus in the axil of rachis and partial inflorescence. The flowers are borne on the third order of branching in the staminate plant but on the second order of the pistillate plants. Each partial inflorescence is in c. four units, c. 3.9 cm in length (staminate plants) and c. 3.3 cm in length (pistillate plants); branches further four to six times. The staminate rachilla is c. 1.8 cm long, with alternately and distichously arranged flowers. The calyx is green with brown tip, c. 1.1 mm; sepal in three, yellowish green; petal in three, cream white, c. 0.8 mm; the pistillode is trilobed but never open, orange; the stamen is c. 2.0 mm with the anther bilobed with powdery yellow pollen, which is dehisced through a lateral slit, filament white almost opaque (Fig. 2E). The pistillate flowers with the calyx green but brown near the margin, c. 0.6 mm long; the petals in three, cream white, c. 1.6 mm; stigma is trilobed and opaque, almost transparent, c. 1.9 mm. The sterile staminate flowers with the calyx green with brown tip, c. 0.4 mm; the sepal in three, cream white, c. 1.0 mm; the petal in three, cream white; staminode in six, opaque, c. 2.0 mm; the pistillode is trilobed but never open, c. 1.2 mm (Fig. 2F). The fruit is (white when mature) round c. 7.3 mm in diameter with the stigma remnant, covered in 11–14 vertical rows of straw-coloured margined scales (Fig. 2G). A summary of plant habits and floral characters of *C. lobbianus* and *C. pygmaeus* is provided in Table 2.

### Floral Biology and Floral Visitors

Several days of direct observations were conducted to establish the start and the end of the anthesis. This was followed by direct observations consisting of four to five hours each day until the entire duration of the anthesis was observed. The total number of flowers per inflorescence was counted. Floral visitors were observed from when flowers were still enclosed in bracts until the end of anthesis. The behaviour of the floral visitors such as interaction with stigma and anthers, feeding on nectar, and the average duration of visit. The floral visitors were further categorised into primary and secondary visitors based on their interactions with the flowers. The mean number of floral visitors was presented based on the total duration of a visit for each species.

### Floral Availability

A total of 13 individuals (seven staminate and six pistillate) for *C. lobbianus* and 138 individuals (55 staminate and 78 pistillate) for *C. pygmaeus* were included in this study. The observation was carried out from 21 September 2021 till 14 January 2022. The sex ratio at two levels (the number of individuals and the number of individuals that flowered during the study period) was tested using binomial tests (Kidyoo & McKey 2012).

### Floral Nectar

The volume and concentration of nectar were measured for both sexes of Forms A and B of *C. lobbianus*. The nectar was collected using a 10 μL microcapillary syringe while the concentration was measured using a handheld Brix refractometer model 45–81 (Bellingham + Stanley) at 30-min intervals. The Brix reading is assumed as sucrose equivalent (Dafni 1992). Nectar sampling was performed during phenology observations and the number of flowers sampled was dependent on the floral availability (Appendix A).

### Pollination

Two treatments were applied: Bagged flower (T1) – partial inflorescence was bagged (using 0.5 mm mesh bags) several days before blooming; Free unbagged (T2) – flowers were left exposed to permit unhindered interactions with floral visitors. The bags were removed after anthesis and several months later the fruit set was registered. To eliminate any possible effect of inherent variation in the fruit set, a different part of each inflorescence was bagged. One to two individuals from each population were selected; partial inflorescences from four individuals were tagged for *C. pygmaeus* while partial inflorescences from three individuals were tagged for *C. lobbianus*. Further details on the samplings are provided in Appendix B.

## RESULTS

### Phenology

The phenology for both forms of *C. lobbianus* is similar for staminate and pistillate plants. The blooming of the staminate flowers for *C. lobbianus* begins at the distal end of the proximal partial inflorescences. However, the flowers of the remaining partial inflorescences bloom randomly. Two to three staminate flowers of a partial inflorescence bloom per day with one to three-day intervals. The stamen and pistillode extension happen simultaneously during blooming. Pollen extrusion begins between 0500 to 1000 (Day 1) and lasts until around 1300, sometimes 1500 (Day 1). Nectar was exuded from near the base of the petals around 1000 (Day 1) till 1300 on the same day. No odour was discernible even directly above the flowers. The flower dehisces around 1500 (Day 1). A partial inflorescence takes c. 8 days to finish blooming while the whole inflorescence takes c. 25 days.

The blooming of pistillate flowers of *C. lobbianus* begins from the distal end of the distal partial inflorescence but the flowering sequence is random within the partial inflorescences. Two to three pistillate flowers of a partial inflorescence bloom per day with one to three-day intervals. The stigma begins to emerge around 0800 to 1000 one day before anthesis. By 0400 to 0700 on the following day (Day 1), the pistillate anthesis begins with the glistening of the surface of the stigma. The stigma is fully-emerged with the recurving petals by 1200 (Day 1). The stigma dries which indicates the end of the anthesis between 1500 to 1700 (Day 2). In the associated dyad counterpart, sterile staminate flowers typically open around 0600 (Day 1) and fall off around 1600 on Day 1. However, 17.85% (5/28) of the staminodes do not open synchronously with their associated pistillate flower. Nectar is exuded from the base of the petals of the sterile staminate flowers from around 0900 (Day 1) till around 1230 on the same day. The blooming lasts about nine days in a partial inflorescence which takes the inflorescence around 19 days to finish blooming.

The blooming of both sexes of *C. pygmaeus* begins from the distal end of the proximal partial inflorescence but the flowering sequence is random within the partial inflorescences. Several partial inflorescences bloom at the same time in older plants of *C. pygmaeus*. Three to four staminate flowers bloom per day with one to two-day intervals. In staminate plants of *C. pygmaeus*, the anthesis of the individual flowers begins from 0830 (Day 1) with some flowers opening much later, at c. 1000. A thin layer of glistening exudate was visible at the base of the petals typically 30 min after anthesis begins. Stamen and pistillode extension happen simultaneously during blooming. No floral scent was detected throughout the anthesis. Anthesis ends by 1500 (Day 1) and the flower is shed by 1800 (Day 1). The blooming lasts about 12 days in a partial inflorescence which takes the inflorescence around 45 days to finish blooming.

**Figure 3:**
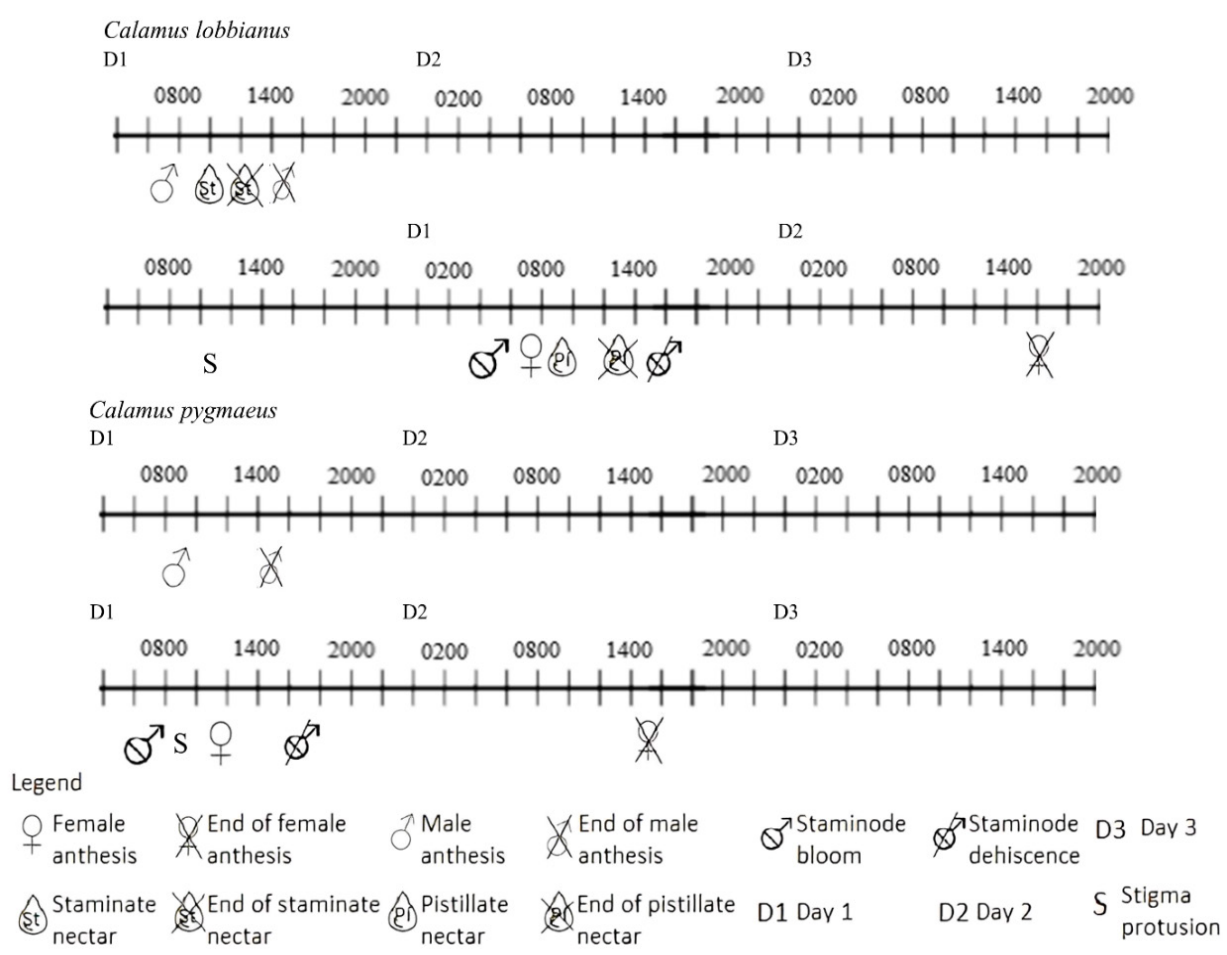
A timeline of phenological events for *C. lobbianus* and *C. pygmaeus*.

Two to three pistillate flowers of *C. lobbianus* bloom per day with one to three-day intervals with at least one or more staminodes are open within the partial inflorescence. The stigma emerges around 0800 (Day 1). The anthesis begins around an hour later, 0900 (Day 1) with the stigmatic surface appearing receptive and white until 1500 (Day 2). The stigmatic surface turns brown towards the evening with the corolla and calyx shed. No floral scent was detected. The sterile staminate flower of *C. pygmaeus* opens around 0800 (Day 1), persists until around 1600 (Day 1) and dehisces by 1700 (Day 1). The base of the petals appears to be glistening, likely to be nectar. The sterile staminate flower blooms randomly, which could be before, during or after the blooming of the associated pistillate flower. The blooming lasts about seven days in a partial inflorescence which takes the inflorescence around 30 days to bloom. The phenological phases are represented in Fig. 3.

### Vegetative Propagation in *Calamus pygmaeus*

In *C. pygmaeus*, vegetative propagation is achieved via the rooting at the tip of inflorescence (Fig. 2H). *C. pygmaeus* displays a population of the same gender within 2 m of each other (2 m is the average length of the inflorescence). Each patch contains 9 to 24 individual plants and sometimes up to 30 individual plants of various developmental stages, with usually one or several individuals in the reproductive stage (Table 3). The rooting at the tip of inflorescence was recorded in 54.54% of the male individuals and 55.12% of the pistillate individuals (Table 3).

### Floral Availability and Operational Sex Ratio

**Figure 4:**
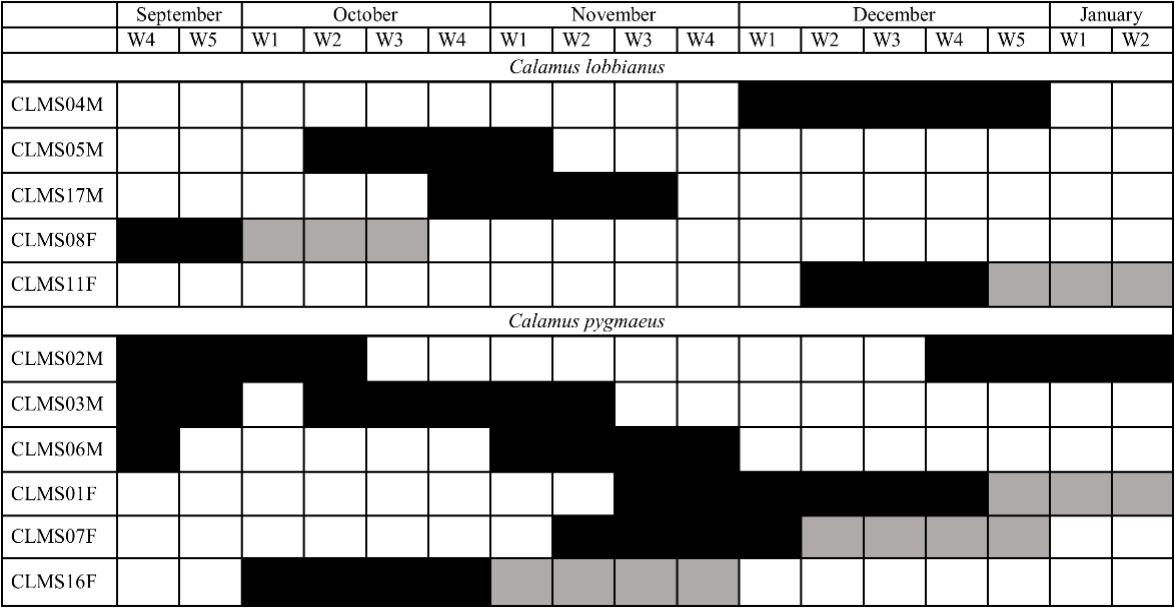
The presence of staminate and pistillate inflorescence for *C. lobbianus* and *C.pygmaeus* from 21 September 2021 until 14 January 2022. W1 = Week 1 and so on; voucher code ending with M is a staminate plant, voucher code ending with F is a pistillate plant; the black box represents blooming, and the grey box represents fruiting.

The presence of staminate and pistillate inflorescence for *C. lobbianus* and *C.pygmaeus* is presented in Fig. 4. Flowering for both species appears aseasonal throughout the observed period although *C. pygmaeus* blooms more frequently as compared to *C. lobbianus*. The sex ratio of flowering individuals was largely unbiased between male and female individuals except during October and December of 2021 for *C. lobbianus* where the male bias was significant (*p* = 0.044)(Table 4).

### Floral Nectar Availability

**Figure 5:**
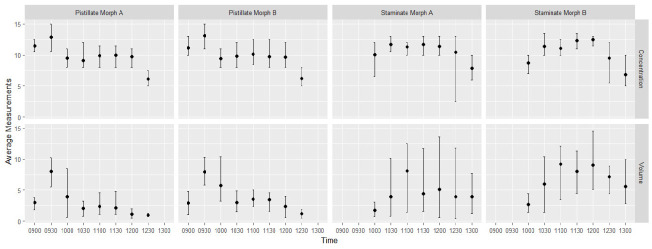
Average volume (μL) and concentration (% in Brix) of pistillate and staminate flowers for Morph A and Morph B of *C. lobbianus* through time with error bars represented by maximum and minimum values.

The volume and concentration of sugar in the nectar varied throughout anthesis for *C. lobbianus* (Forms A and B) as shown in Fig. 5. In the staminate flower of Form A, the volume and concentration started from 1.7 μL and 10.1% and peaked at 8.1 μL and 11.3% (c. 1100 Day 1) (Table 5). The associated sterile staminate flower of the pistillate plant produced 2.7 μL amount of 8.8% (concentration) till peak at 8.0 μL and 12.9% (concentration) (c. 0930 Day 1). A similar pattern was observed for both sexes of Form B plants of *C. lobbianus*.

### Floral Visitors

**Figure 6:**
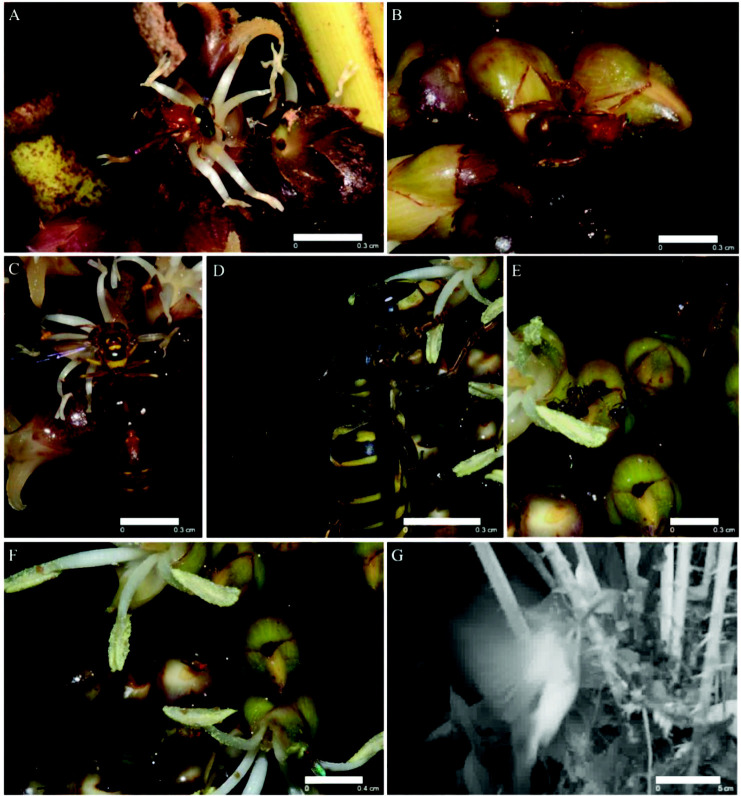
Floral visitors of *C. lobbianus*. (A) *Tetragonula melanocephala*, black-headed stingless bee; (B) *T. melina*, orange stingless bees; (C) *Liostenogaster* sp., hover wasp; (D)*Stenodyneriellus* sp., potter wasp; (E) Unidentified ant species; (F) *Drosophila* sp., fruit flies and floral mites; and (G) *Arachnothera* sp., spiderhunter. Scale bars are included.

**Figure 7:**
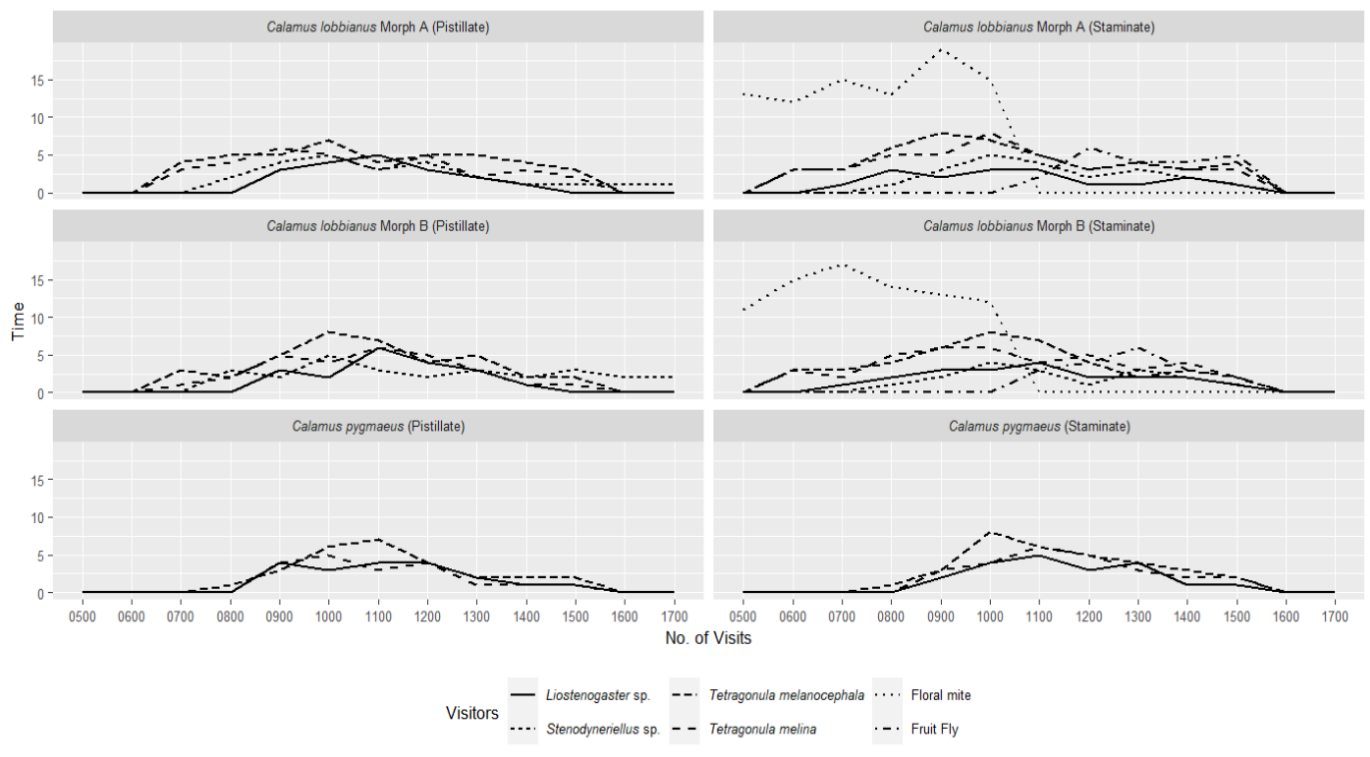
Average number for each species of visitor throughout visiting duration for both morphs of *C. lobbianus* and *C. pygmaeus*.

The floral visitors observed for *C. lobbianus* were quite diverse and consisted of several different taxa, black-headed stingless bee (*Tetragonula melanocephala*) (Fig. 6A), orange stingless bee (*T. melina*) (Fig. 6B), hover wasp (*Liostenogaster* sp.) (Fig. 6C), potter wasp (*Stenodyneriellus* sp.) (Fig. 6D), black ants (Formicidae) (Fig. 6E), floral mites (Acaridae) (Fig. 6F), fruit flies (*Drosophila* sp.) (Fig. 6F) and spiderhunter (*Arachnothera* sp.) (Fig. 6G). The visiting time of the wasps and stingless bees was similar for the staminate flowers, starting from early morning around 0700 (Day 1) to about 1500 (Day 1) (Table 6; Fig. 7). Both species of stingless bees have similar behaviours where they feed on nectar and collect pollen. Both hover and paper wasps were observed to not actively collect pollen but mainly fed on the nectar, but the potter wasp is larger than the flowers which sometimes allows the anthers to brush against its body. However, it is rare for pollen to be seen stuck onto its head or thorax. A nest of black ants was observed to linger around the inflorescence during the bud formation and bloom to feed on the nectar without contacting the anthers (observed only on one occasion). On several observations, floral mites were seen attached to the visiting insects and carried to another flower throughout anthesis of both sexes. The mites shifted within and between the blooming flowers to feed on pollen and nectar. Fruit flies were always present and occasionally observed to feed on the floral nectar of both sexes. Once, a spiderhunter was observed to visit the flowers around 1200 to feed on the (Day 1) nectar, however, the long beak of the bird did not allow the anther to be in contact with the head and the spiderhunter is thus considered a visitor. Visitors of the pistillate flowers of *C. lobbianus* were similar excluding fruit flies and floral mites. The floral visitors were found during the first day of anthesis (Table 6). Both species of stingless bees were observed to transfer the pollen onto the stigma and thus are considered as possible pollinators. Both wasps aimed for the nectar in the staminodes and seldom seem to be in contact with the stigma and thus are considered a secondary pollinator.

**Figure 8:**
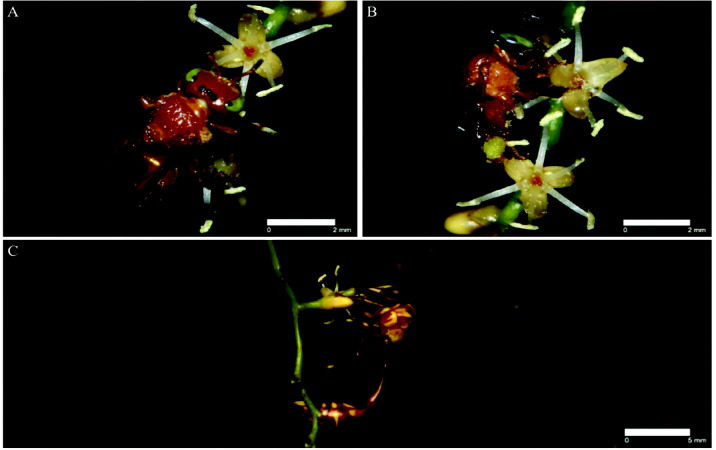
Floral visitors of *C. pygmaeus*: (A) *Tetragonula melina*, orange stingless bees; (B) *T. melanocephala*, black-headed stingless bee; (C) *Liostenogaster* sp., hover wasp. Scale bars are included.

The orange stingless bee (*Tetragonula melina*) (Fig. 8A), black-headed stingless bee (*T. melanocephala*) (Fig. 8B), and the hover wasp (*Liostenogaster* sp.) (Fig. 8C) visited the flowers of *C. pygmaeus* from 0830 until 1430 (Day 1) for both staminate and pistillate flowers. The hover wasp and both stingless bees continue to visit on Day 2 from 0830 until 1430 in pistillate plants. Both stingless bees usually landed on the anther, collected pollen and sometimes headed into the cavity to search for nectar. During its visit to the pistillate flower, both stingless bees behaved similarly but remained slightly longer. The hover wasps generally headed into the floral cavity to search for nectar in both staminate and pistillate flowers. However, owing to the lack of pollen on its body, the hover wasp is considered a secondary pollinator. Several species of ants were observed to visit the flowers on rare occasions but are not considered pollinators.

There is no significant difference (*p* > 0.05) in the number of visitors and visit duration on of all visitors except for the visit duration of hover wasps, fruit flies and floral mites between the staminate and pistillate plants of Forms A and B of *C. lobbianus* (Table 6). There is also no significant difference on the floral visitors between the different forms of *C. lobbianus*.

### Breeding Mechanism

Pollination experiment on *C. lobbianus* indicates that apomixis is likely to exist as the percentage of fruit set for bagged treatment was lower at 4.77%, 8.33% and 7.68% as compared to unbagged treatment at 21.05%, 19.05% and 16.67% although not statistically significant. However, a seed viability test on apomictic fruit was not performed.

The fruit set was recorded in all bagged treatments at three populations which indicates that apomixis is present in *C. pygmaeus* (Table 7). However, the results showed no significant difference between both treatments for Populations 6 and 1 which is likely owing to the pistillate plants showing poor fruiting success as compared to other populations. In Population 5 of *C. pygmaeus*, the fruit set percentage of unbagged treatment was significantly higher than the bagged treatment which differs from the other two populations.

## DISCUSSION

### Pollinators, Visitors and Floral Rewards

A wide array of visitor assemblage was reported on *Calamus* mainly Acaridae, Coleoptera, Diptera, Hymenoptera, Lepidoptera and Thysanoptera (Alloysius 1999; Bøgh 1996; Dransfield 1979b; Kidyoo & McKey 2012; Kitching *et al*. 2007; Lee 1995). However, pollinators of *Calamus* are mainly Hymenopterans such as stingless bees, honeybees and some Vespid wasps (Bøgh 1996; Kidyoo & McKey 2012; Lee 1995). Lee (1995) reported a wide array of visitors for *C. subinermis* and *C. caesius*, however, most were recorded in very low abundance with the highest recorded from Hymenopterans similar to this study. Some Coleopterans such as nitidulid beetles and weevils are also considered potential pollinators (Dransfield 1979a; 1979b). The stingless bees are the most possible pollinators for both *Calamus* species in this study with *C. lobbianus* being pollinated by more than one species of stingless bees. The stingless bees were observed to have pollen attached onto their body and transfer it onto the pistillate flowers during their foraging activities. *Liostenogaster* sp. and *Stenodyneriellus* sp. as pollinators in *Calamus* have yet to be reported previously; thus, this is the first record, however, members of this family are known to be pollinators in many other plant families (Bøgh 1996; Kidyoo & McKey 2012). The flowers of *C. pygmaeus* are not particularly showy as compared to *C. lobbianus* with relatively small flowers and do not produce scent nor nectar in droplets. The lack of droplets of nectar has also been previously observed in *C. castaneus* (Kidyoo & McKey 2012). *C.lobbianus* on the other hand has large showy flowers and complemented byits production of nectar is capable of attracting a myriad of visitors.

A factor that may attract pollinators is that some palms initially attract visitors with colour vision through the mass flowering of orange-yellow or white flowers without other major anthecological adaptations (Kiew & Muid 1989; Listabarth 2001; Mergos & Yang 2022), which may serve to confuse the visitors for other species that generally offers rewards with higher nutritional values. The role of the staminate and staminode flowers in this *C. pygmaeus* appears to function as attractants for potential pollinators for pollen transfer which has been observed in several other palms (Kidyoo & McKey 2012; Uhl & Moore 1977). The pistillode found on the staminate flowers in the case does not appear to serve any obvious functions apart from being a remnant of a perfect flower. The anthers protrude in all directions and are capable of depositing pollen on any insect of suitable size that crawls around the flowers and the same can be said about the stigma which covers a large area of the pistillate flower. The flowers of *Calamus* have most likely evolved to take advantage of generalist species of pollinators which in this case does not appear seasonal throughout the observation period. The availability of flowers does not suggest seasonality as well since flowers of both staminate and pistillate are available continuously or at the least intermittently throughout the observation period (Fig. 4).

The flowers within one inflorescence often bloom in distinct sequences which may be associated with the pollination mechanism. Basipetal maturation is a process in which the development of plant tissues or organs proceeds from the apex to the base, while acropetal maturation is the opposite process, in which development proceeds from the base to the apex (Dafni 1992). Henderson (2002) suggested a possible correlation between basipetal floral maturation and beetle pollination while acropetal floral maturation in triads/flowers with bee, fly and wasp in Arecaceae. The flowering sequence in *C. lobbianus* is acropetal in staminate plants but basipetal in pistillate plants while *C. pygmaeus* is acropetal entirely. *Calamus lobbianus* is likely to be another exception similar to *Licuala peltata* Roxb. ex Buch.-Ham. (Barfod *et al*. 2003). Basipetal or acropetal maturation may alter the morphology and physiology of flowers, such as their size, shape, colour, scent, nectar production and pollen viability, which may also influence pollinator attraction and efficiency. These morphological and physiological differences are directly influenced by the production of secondary metabolites such as phenolics, terpenes and alkaloids (Taiz & Zeiger 2010).

It is likely that *C. lobbianus* and *C. pygmaeus* do not strictly rely on the observed primary visitors for pollination but instead have low taxonomic pollinator specificity and are pollinated by a mixed species guild as suggested by Listabarth (2001). Previous pollination works also suggest mixed guild pollination similar to our results (Alloysius 1999; Bøgh 1996; Lee 1995). The observed visitors could be purely owing to their foraging behaviours and nest proximity to the population. Several groups of flies including the fruit flies are always present on the inflorescence, they are, however, generalist of all kinds of flowers and likely of little consequence for pollination in palms, except perhaps in the case of *Geonoma cuneata* var. *sodiroi* in which Borchsenius (1997) suggested that drosophilid flies are the primary pollinator. A more comparative view can be generated by sampling on a larger geographic scale to identify if there is any degree of specialisation within the plant-pollinator interactions which is free from the variables generated by local settings which are currently still not available.

### Sex Ratio Bias

The operational sex ratio is the ratio of receptive staminate to receptive pistillate flowers in a population at any time (Kidyoo & McKey 2012). Flowering occurs year-round for both species, thus ideally there should be staminate and pistillate flowers available at all times, however, pistillate plants in the bloom of *C.lobbianus* occur less frequently compared to staminate individuals. While there are statistically significant biases observed in *C. lobbianus*, it should be noted that the sample size of *C. lobbianus* is small (seven staminate and six pistillate plants)owing to the difficulty in encountering more flowering populations as compared to *C.pygmaeus* (55 staminate and 78 pistillate plants). In *C. lobbianus*, the male-biased scenario provides a ready supply of pollen. The frequent flowering of the staminate plants likely trains the insect visitors to always visit the staminate plants and consequently carry the pollen regardless of the insects visiting the pistillate plants of the same species which in turn increases the likelihood of depositing pollen onto the receptive stigmas. This method works as the main insect visitors were mainly Hymenopterans which have been recorded to develop preferences and repeatedly visit the same plants (Fohouo *et al*. 2008; Mattu *et al*. 2012; Sushil *et al*. 2013). In *C. pygmaeus*, although no sex ratio bias was detected the high number of staminate plants blooming also provided a steady supply of pollen for the pistillate plants. *Calamus castaneus* has also been reported to be aseasonal, flowering through year-round with staminate plants flowering more frequently compared to pistillate (Kidyoo & McKey 2012; Mohd Rusdi *et al*. 2022).

### Unique Mode of Vegetative Reproduction in *Calamus*

Currently, there are at least eight species known to be capable of forming new plants at the apex of inflorescences within this genus; four from the Indochinese region (*C. castaneus, C. kampucheansis, C. parvulus* A. J. Hend. & N. Q. Dung and *C.rhabdocladus* Burret) and four from Sunda region (*C. gaharuensis, C. ingens*(J. Dransf.) W. J. Baker, *C. pygmaeus* and *C. ruptiloides* A. J. Hend.) (Dransfield 1992; Henderson 2020). While this trait is spread across Southeast Asia, it is relatively uncommon within the genus (Henderson 2020). The only other genus within Calameae with this trait is *Salacca* (Henderson 2009). It is probable that this trait was once more widespread within the genus but has since been outselected and is now a remnant trait. This trait also shows up only in certain populations or individuals and is not common within the species as in the case of *C. castaneus* and *C. rhabdocladus* possibly as a form of mutation (Henderson 2020; Mohd Rusdi *et al*. 2022; Ruppert *et al*. 2012). *C. dianbaiensis* C. F. Wei has a similar trait but instead, roots at the shoot apex after growing vertically up to 10 m, bends over and roots when the shoot touches the ground to produce new plants (Henderson 2020).The clustering habit observed in *C. javensis* Blume where clusters are established through adventitious roots on the aerial stem that can attach to the soil after the stem falls is very similar to the clustering in *C. pygmaeus*. While the method of clustering differs, they ultimately create a colony of clones within a certain radius. Genetic testing was not performed in this study, however, the individuals within each colony of *C. pygmaeus* were of the same sex and in some cases the tip of the inflorescence was still attached. Watanabe *et al*. (2006) suggest the clonality in *C. javensis* is very likely a growth strategy to increase the size of the genet rather than a dispersion-propagation strategy to expand the habitat and that the separate clusters are established by seed recruitment with seed obtained from nearby clusters. Given the highly similar clustering pattern, *C. pygmaeus* most likely also evolved to adopt the same strategy.

### Apomixis in *Calamus*

In a previous study, it was revealed that *C. longisetus, C. peregrinus* and *C. rudentum* are not capable of apomixis (Bøgh 1996) while other members of the family (i.e., *Chamaedorea* Wild., *Howea* Becc., *Attalea funifera* Mart. ex Spreng.), showed reduced and sometimes no fruit set in bagged pollination experiments (Otero-Arnaiz & Oyama 2001; Rios *et al*. 2014; Savolainen *et al*. 2006; Voeks 2002). It should be noted that while fruit set has occurred in bagged flowers, it is still possible for pollen transfer to occur by smaller insects that may have slipped through the fine mesh or pollen deposited on the wall of the mesh beg which came in contact with the stigma. Both *C. lobbianus* and *C. pygmaeus* are unlikely to undergo apomixis or at least in a reduced form which is in congruence with the other members of the family. While results from the pollination experiments suggest apomictic behaviour, apomixis is a rare trait within the family Arecaceae and is not widespread unlike in large families such as Asteraceae, Poaceae and Rosaceae (Hojsgaard *et al*. 2014).

## CONCLUSIONS

*C. lobbianus* and *C. pygmaeus* while similar in phenology, exhibit stark differences in their floral characteristics and attractants, leading to distinct reproductive strategies. This contrast is directly reflected in the visitor assemblage which subsequently reveals their breeding strategy. While both species belong to the same genus, *C. lobbianus* reproduces mainly through outbreeding sexual reproduction while *C. pygmaeus* adopts a unique reproductive approach, combining sexual and asexual methods by generating plantlets at the inflorescence tip. While this study does not delve into it, exploring the impacts of these diverse reproductive strategies on population genomics and gene flow, especially in *C. pygmaeus*, could yield intriguing insights.

## Figures and Tables

**Table 1:** Vegetative and reproductive differences between both forms in staminate and pistillate plants of *C. lobbianus*.

	Form A			Form B		

	Staminate	Pistillate	Sterile staminate	Staminate	Pistillate	Sterile staminate
Petiolar spine	c.14.6 mm	c.9.8 mm	-	c. 11.3 mm	c.8.9 mm	-
Calyx	Light green with a brown margin and white lateral ribs (c. 5.5 mm in height).	Light green with white dots, reddish-purple vertical lines and a brown margin (c. 5.7 mm long).	Light green (c. 4.9 mm).	Maroon with light maroon lateral ribs (c. 5.2 mm in height).	Maroon on the top half, light green on the bottom half with light green dots and vertical lines (c. 4.9 mm).	Maroon on the top half, light green on the bottom half with light green dots and vertical lines (c. 4.7 mm).
Petals	White on the lateral ribs, green at the distal half and light green at the proximal half with white dots, internally white at the distal half and light green at the proximal half (c. 7.1 mm).	Light green with white dots, reddish-purple vertical lines and a brown margin (c. 5.7 mm long).	Light green with white dots, reddish-purple vertical lines and a brown margin (c. 5.5 mm long).	Petals are pink with a white margin and light brown lateral ribs externally, internally light pink (c. 6.8 mm).	Entirely cream white (c. 7.4 mm).	Entirely cream white (c. 7.3 mm).
Sepals	-	Yellowish green(c. 6.3 mm).	Yellowish green (c. 6.2 mm).	-	Maroon on the top half, light green on the bottom half with light green dots and vertical lines (c. 6.4 mm).	Maroon on the top half, light green on the bottom half with light green dots and vertical lines (c. 6.1 mm).

**Table 2:** A summary of plant habits and floral characters of *C. lobbianus* and *C. pygmaeus* with data presented as an average (range) unit.

	*C. lobbianus*	*C. pygmaeus*
Habit	Solitary	Clustering
Stem length (m)	0.6 (0.4–1.2) m	0.6 (0.3–0.9) m
Vertical height (m)	1.5 (1.3–1.8) m	1.2 (0.9–1.7) m
Inflorescence length ♀ (cm)	26.5 (21.3–31.7) cm	2.5 (2.1–2.7) m
Inflorescence length ♂ (cm)	31.5 (27.4–35.6) cm	2.7 (2.4–2.9) m
Partial inflorescence ♀ length (cm)	11.5 (9.7–17.2) cm	5.7 (5.1–6.2) cm
Partial inflorescence ♂ length (cm)	18.2 (15.9–25.5) cm	6.2 (5.5–7.4) cm
Colour of tepals	Light green with brown margin/ Pink with white margins	Cream white
No. of partial ♀ inflorescence	2 (1–3)	4 (3–7)
No. of partial ♂ inflorescence	2 (1–3)	4 (3–6)
No. of rachis per partial ♀ inflorescence	2 (2–3)	5 (4–6)
No. of rachis per partial ♂ inflorescence	2 (2–4)	5 (2–7)
No. of dyads per ♀ rachis	19 (15–30)	7 (5–9)
No. of flowers per ♂ rachis	41 (32–47)	9 (7–13)
Flowering duration of ♀ inflorescence	19 (18–27)	30 (28–33)
Flowering duration of ♂ inflorescence	25 (21–30)	45 (37–51)
Flowering duration of partial ♀ inflorescence	9 (6–10)	7 (6–9)
Flowering duration of partial ♂ inflorescence	8 (7–10)	12 (10–14)

**Table 3:** Individuals of *C. pygmaeus* with rooted inflorescences.

Staminate/Pistillate	Cluster	No. of individuals	Rooted inflorescence	Rooting percentage (%)
Staminate	Cluster 2	9	4	44.44
	Cluster 3	10	3	30.00
	Cluster 4	21	15	71.43
	Cluster 7	15	8	53.33

Total		55	30	54.54

Pistillate	Cluster 1	13	9	69.23
	Cluster 5	15	10	66.67
	Cluster 6	14	7	50.00
	Cluster 8	24	14	58.33
	Cluster 9	12	3	25.00

Total		78	43	55.12

**Table 4:** Binomial test results in the sex ratio of flowering individuals and the operational sex ratio (OSR) expressed as the ratio of flowering staminate to pistillate plants (staminate: pistillate) of *C. lobbianus* and *C. pygmaeus* (α = 0.05).

Month	Species	Staminate/Pistillate	*p*-value	OSR
October	*C. lobbianus*	Staminate	0.044	2:0
		Pistillate	0.302	
	*C. pygmaeus*	Staminate	0.218	6:5
		Pistillate	0.128	
November	*C. lobbianus*	Staminate	0.302	1:1
		Pistillate	0.265	
	C.pygmaeus	Staminate	0.182	10:11
		Pistillate	0.141	
December	*C. lobbianus*	Staminate	0.044	2:1
		Pistillate	0.265	
	*C. pygmaeus*	Staminate	0.182	5:6

**Table 5:** Volume (μL) and concentration measured in Brix (%) using sucrose equivalence for staminate (♂) and sterile staminate (⚦) plants of *C. lobbianus* for Forms A and B.

Volume/ Concentration	0900	0930	1000	1030	1100	1130	1200	1230	1300
*Calamus lobbianus* A ♂									
Volume (μL)	-	-	1.7 (SE ± 0.40)	3.9 (SE ± 1.34)	8.1 (SE ± 2.12)	4.4 (SE ± 1.56)	5.1 (SE ± 1.97)	3.9 (SE ± 1.81)	3.9 (SE ± 1.00)
Concentration (% in Brix)	-	-	10.1 (SE ± 0.78)	11.7 (SE ± 0.40)	11.3 (SE ± 0.31)	11.7 (SE ± 0.44)	11.4 (SE ± 0.49)	10.4 (SE ± 1.60)	7.8 (SE ± 0.61)
*Calamus lobbianus* A ⚦									
Volume (μL)	3.0 (SE ± 0.28)	8.0 (SE ± 0.75)	3.9 (SE ± 1.19)	2.0 (SE ± 0.34)	2.4 (SE ± 0.61)	2.1 (SE ± 0.57)	1.1 (SE ± 0.22)	0.9 (SE ± 0.10)	-
Concentration (% in Brix)	11.5 (SE ± 0.29)	12.9 (SE ± 0.68)	9.5 (SE ± 0.45)	9.1 (SE ± 0.62	9.9 (SE ± 0.54)	10.0 (SE ± 0.53)	9.9 (SE ± 0.48)	6.2 (SE ± 0.38)	-
*Calamus lobbianus* B ♂									
Volume (μL)	-	-	2.7 (SE ± 0.48)	6.00 (SE ± 1.24)	9.1 (SE ± 1.54)	7.9 (SE ± 1.41)	9.0 (SE ± 1.62)	7.1 (SE ± 0.69)	5.6 (SE ± 1.00)
Concentration (% in Brix)	-	-	8.8 (SE ± 0.51)	11.4 (SE ± 0.51)	11.1 (SE ± 0.35)	12.3 (SE ± 0.40)	12.5 (SE ± 0.26)	9.5 (SE ± 0.91)	6.8 (SE ± 0.84)
*Calamus lobbianus* B ⚦									
Volume (μL)	2.9 (SE ± 0.65)	7.9 (SE ± 0.74)	5.7 (SE ± 1.17)	3.0 (SE ± 0.53)	3.5 (SE ± 0.49)	3.5 (SE ± 0.49)	2.3 (SE ± 0.57)	1.2 (SE ± 0.21)	-
Concentration (% in Brix)	11.2 (SE ± 0.46)	13.2 (SE ± 0.62)	9.4 (SE ± 0.47)	9.8 (SE ± 0.71)	10.2 (SE ± 0.60)	9.8 (SE ± 0.74)	9.7 (SE ± 0.63)	6.3 (SE ± 0.42)	-

**Table 6:** Visiting period, mean number and duration of visit for staminate (♂) and pistillate (♀) plants of *C. lobbianus* and *C. pygmaeus*; value represented as min(average)max for Day 1 (D1) and Day 2 (D2). Significant results between forms of *C. lobbianus* are represented by * (*p* > 0.05).

Species	Visiting period		Average visitor		Duration		Visitation rate (*p*-value)		Visit duration (*p*-value)	

	♂	♀	♂	♀	♂	♀	♂	♀	♂	♀

	*Calamus lobbianus* Form A						*t*-test results in *p*-value between Forms A and B			

*Liostenogaster* sp.	0700–1500 (D1)	0830–1400 (D2)	1(2)3	1(3)5	1.2 sec	4.3 sec	0.47	0.86	0.00*	0.21
*Stenodyneriellus* sp.	0800–1500 (D1)	0800–1700 (D2)	1(3)5	1(2)5	5.5 sec	6.3 sec	0.45	0.60	0.24	0.14
*Tetragonula melina*	0600–1500 (D1)	0700–1500 D2)	3(4)7	2(4)6	5.7 sec	6.1 sec	0.78	0.50	0.08	0.49
*Tetragonula melanocephala*	0600–1500 (D1)	0700–1500 (D2)	3(5)8	3(5)7	6.3 sec	6.8 sec	0.66	0.60	0.09	0.54
*Drosophila* sp.	1100–1500 (D1)	-	2(4)6	-	5.6 sec	-	0.55	-	0.02*	-
Floral mite	0500–1000 (D1)	-	13(15)19	-	31.5 min	-	0.55	-	0.00*	-

	*Calamus lobbianus* Form B									

*Liostenogaster* sp.	0700–1500 (D1)	0830 (D1)–1400 (D2)	2(2)4	1(2)6	2.8 sec	3.8 sec				
*Stenodyneriellus* sp.	0800–1500 (D1)	0800 (D1)–1700 (D2)	1(2)4	2(3)5	6.2 sec	5.6 sec				
*Tetragonula melina*	0600–1500 (D1)	0700 (D1)–1500 D2)	2(5)6	1(3)6	4.6 sec	6.5 sec				
*Tetragonula melanocephala*	0600–1500 (D1)	0700 (D1)–1500 (D2)	2(4)8	2(4)8	7.3 sec	7.3 sec				
*Drosophila* sp.	1100–1500 (D1)	-	2(3)6	-	4.5 sec	-				
Floral mite	0500–1000 (D1)	-	11(13)17	-	27.6 min	-				

	*Calamus pygmaeus*									

*Liostenogaster* sp.	0830–1430 (D1)	0830 (D1)–1430 (D2)	1(3)4	1(3)4	1.5 sec	2.5 sec				
*Tetragonula melina*	0830–1430 (D1)	0830 (D1)–1430 (D2)	2(3)6	1(3)5	3.6 sec	4.5 sec				
*Tetragonula melanocephala*	0830–1430 (D1)	0830 (D1)–1430 (D2)	2(4)8	2(3)7	5.2 sec	6.7 sec				

**Table 7:** Results from pollination experiment on *C. pygmaeus* and *C. lobbianus*.

	T1	T1 fruit set	T2	T2 fruit set	Chi-square	*p*-value
	*Calamus pygmaeus*					

Population 6	2/78	2.56%	6/68	8.82%	2.748	0.0974
Population 5	2/19	10.53%	6/8	75.00%	11.224	0.0008
Population 1	3/12	25.00%	2/14	14.29%	0.2143	0.498

	*Calamus lobbianus*					

CLMS11F	1/22	4.77%	4/19	21.05%	0.7748	0.1245
CLMN08F	1/24	4.17%	4/21	19.05%	0.4489	0.2386
CLMS018F	2/26	7.69%	5/30	16.67%	0.3306	0.3155

*Notes*: T1 = bagged flower, T2 = free unbagged; Chi-square (χ^2^) and *p*-value is calculated between results from T1 and T2 where α = 0.05.

## APPENDICES

### Appendix A Total number of individuals, inflorescences and flowers observed for *C. lobbianus* and *C. pygmaeus*. Code CLMS represents individuals of *C. lobbianus* sampled for nectar volumetric and concentration study (both measurements were taken in tandem)

[Table t8-tlsr_35-3-185]

**Table t8-tlsr_35-3-185:**

Species/Code		Form	Individuals	Inflorescences	Flowers	Observational period
*Calamus lobbianus*	♂	A	3	4	32	29/4/21–8/5/21
	♀	A	2	4	28	3/12/21–26/4/22
*Calamus pygmaeus*	♂	B	3	4	14	1/4/21–7/5/21
	♀	B	2	3	17	1/4/21–7/5/21
CLMS02M	♂		-	-	16	26/10/21–
						12/11/21
CLMS03M	♂		-	-	24	3/5/21–10/5/21
CLMS01F	♀		-	-	21	7/12/22–18/12/22
CLMS07F	♀		-	-	19	15/6/22–26/6/22

### Appendix B Pollination experiments conducted on three separate populations for *C. pygmaeus* and three individuals for *C. lobbianus*

[Table t9-tlsr_35-3-185]

**Table t9-tlsr_35-3-185:**

Species	Pop.	Voucher code	Unbagged		Bagged	

			Inflor. code	Duration	Inflor. code	Duration
*C. pygmaeus*	Pop 6	CLMS06F	CT001	17/10/21–3/11/21	AP001	3/10/21–21/11/21
*C. pygmaeus*	Pop 6	CLMS06F	CT002	3/10/21–21/10/21	AP002	6/10/21–26/10/21
*C. pygmaeus*	Pop 5	CLMS05F	CT003	15/11/21–3/11/21	AP003	12/11/21–3/11/21
*C. pygmaeus*	Pop 1	CLMS01F	CT004	18/12/21–6/1/22	AP004	27/11/21–18/12/21
*C. lobbianus*	-	CLMS011F	CT005	9/12/21–21/12/21	AP005	1/5/2022–19/5/22
*C. lobbianus*	-	CLMS008F	CT006	13/12/21–4/1/22	AP006	2/6/2022–9/7/22
*C. lobbianus*	-	CLMS018F	CT009	18/3/22–21/4/22	AP009	26/6/22–25/7/22
